# Reported family burden of schizophrenia patients in rural China

**DOI:** 10.1371/journal.pone.0179425

**Published:** 2017-06-19

**Authors:** Yu Yu, Zi-wei Liu, Bing-wei Tang, Mei Zhao, Xi-guang Liu, Shui-yuan Xiao

**Affiliations:** 1 Department of Social Medicine and Health Management, Xiangya School of Public Health, Central South University, Changsha, Hunan province, China; 2 Hospital evaluation office of Xiangya hospital, Central South University, Changsha, Hunan province, China; University of Texas Health Science Center at San Antonio Cancer Therapy and Research Center at Houston, UNITED STATES

## Abstract

We aim to assess the level of family burden of schizophrenia patients and identify its predicting factors in a rural community sample of China. A sample of 327 primary caregivers was recruited through a one-stage cluster sampling in Ningxiang County of Hunan province, China. Family burden was assessed using the Family Burden Interview Schedule (FBIS) of Pai and Kapur. Our results showed that the mean score of FBIS was 23.62±9.76 (range, 0–48), with over half (52%) caregivers reported their family burden being moderate and severe. Among the six domains of family burden, financial burden (76%) was the commonest burden, while disruption of family interactions (37%) was the least mentioned. A multivariate analysis of family burden revealed that patient being admitted for over 3 times, caregiver being female, having a middle school education, and with additional dependents, as well as higher care network function were positive predictors of family burden, while higher patient function and family function, and increasing patient age were negative predictors of family burden.

Intervention to decrease family burden may be best served by improving family function and exploring alternative care model instead of hospitalization.

## Introduction

Globally, schizophrenia is a debilitating, persistent psychiatric disorder that affects approximately 3–6. 6 of 1000 persons [1]. The World Health Organization [2] estimated that there are more than 21 million people suffering schizophrenia worldwide. It has been listed as the 8th leading causes of disability-adjusted life years in the 15 to 44 age group [3], and has extolled significant costs to the patients, the family and the society at large [4]. The past 50 years has witnessed a worldwide shift in psychiatric care from hospital-based to community-based (de-institutionalization) [5], with families providing the majority of care and shouldering the bulk of burden [6]. In western countries, about 25%–50% of schizophrenia patients depend on families, while in Asian countries, about 70% patients are cared for by their families [7–9]. In addition, over 80% of individuals with a schizophrenic family member have been documented to suffer from tremendous burden in various aspects including economy, social activities, physical and mental health [10].

Although China has not experienced de-institutionalization, the Chinese culture is deeply rooted in Confucianism that advocates for family harmony and integrity, with special focus on caring for the impaired family members [9, 11]. Family members of schizophrenic patients in China often take on multiple roles in caring for their sick relatives, which results in considerable burden to the family with physical, mental, social and financial impacts on family members [12, 13]. A moderate to high degree of burden has been reported by many studies in China [12, 14].

Previous research suggests that a range of factors are associated with family burden, which can be classified into three major categories: disease-related factors, demographic factors and social psychological factors, as described below.

### Disease-related factors

The association between disease-related factors and family burden has been extensively researched in the literature, with patient function, duration of illness and treatment being the most frequently studied factors. For patient function, although contradictory results have been reported as regard to whether positive or negative symptoms are more burdensome [10, 15–17], consistency exists in that less severe symptoms and higher level of function results in lower family burden [15, 16, 18–20]. For duration of illness, the majority of study documented a decline in family burden over time [20–22], yet some studies found no effect of time [18]. For treatment, neither length of treatment nor type of treatment has been found to affect family burden [22–24].

### Demographic factors

Demographics of both patients and caregivers have also received abundant attention as influencing factor of family burden. Although numerous studies have yielded equivocal results regarding the association between family burden and age [14, 25, 26], gender [27–29], and kinship [21, 30] of patients and caregivers, a growing body of research congruously indicated that family burden decreases with increasing socioeconomic status [14, 31, 32].

### Social psychological factors

As for social psychological factors, social support, family function, and family coping have been the most reported significant factors predicting family burden, with consistent evidence showing that better social support[4, 33–36], higher level of family function [37–39], as well as positive coping styles [20, 40] were associated with decreased family burden.

The bulk of past studies were limited by a convenience sampling mostly from hospitals and lacking of exhaustive exploration of all the influencing factors of the above-mentioned categories. The present study aims to fill in the knowledge gap by investigating the level of family burden of schizophrenia patients in a representative rural Chinese community sample, and exploring the predicting factors by including representative variables from each of these three categories in our data analysis.

## Methods

### Participants

**Fig 1:**
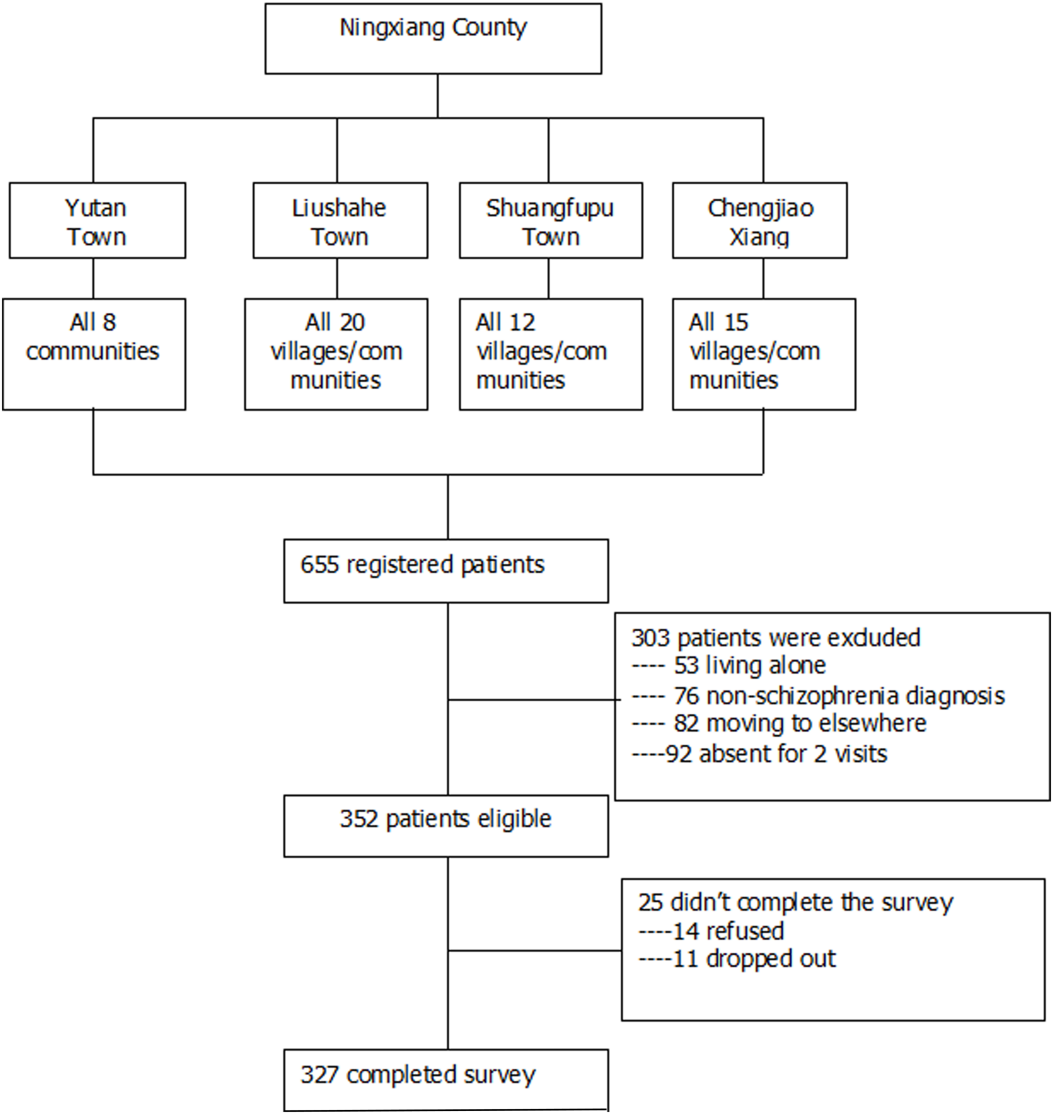
Flowchart of participant enrollment.

Schizophrenia is one of the major mental illnesses included into the management of the ‘Central Government Support for the Local Management and Treatment of Severe Mental Illnesses Project’, also named as ‘686 Program’ due to its first financial allotment of 6.86 million Renminbi($829 000) in 2004[41]. The 686 Program is China’s largest demonstration project in mental health service aimed at integrating hospital and community services for serious mental illness [42–44]. By the end of 2010, the system has registered a total of 280 000 persons with serious mental illnesses [42]. In Ningxiang County, over 3000 patients with serious mental illness have been registered and followed up by the community/village doctors, of whom over 90% were schizophrenia patients. We directly recruited family caregivers of schizophrenia patients for this study from 686 Program through a one-stage cluster-sampling of households (Fig 1).

Ningxiang County is administratively divided into 25 towns and 8 xiangs. Three towns (Yutan, Liushahe, Shungfupu) and 1 xiang(Chengjiao) were firstly randomly selected as the study districts. Then all communities and villages of each township were selected as the study samples, leading to a total sampling frame of 55 communities/villages that were representative of the rural populations in Ningxiang, in terms of geography, socio-demographics, mental health care access and outreach activities. In each community/village, one primary family caregiver of every registered schizophrenia patient was enrolled as the target population. 352 primary family caregivers were selected as the final sample. The primary family caregiver in each family was self-identified as the one who has spent the most time with and provided the most care to the patient for the past two years. Inclusion criteria for this study included: 1) patient being registered in 686 Program; 2) patient fulfilling the Chinese Classification of Mental Disorders-3(CCMD-3) or the International Classification of Diseases-10 (ICD-10) criteria for schizophrenia; 3) the primary caregiver is living with the patient and has taken the most responsibility of caring, with full understanding about the situation of both the patient and the family; 4) primary caregiver older than 16 years old 5) primary caregiver is able to understand and communicate. Exclusion criteria included: 1) patient with diagnosis other than schizophrenia such as depression and epilepsy; 2) patient living alone; 3) primary family caregivers having serious physical or mental illness that are unable to communicate. The response rate was 93%, resulting in a sample of 327 individuals who participated in face-to-face interview for the survey and completed all basic information of the questionnaire, among whom 24 individuals left the FBIS scale blank, leading to an effective rate of 93% for the FBIS.

Demographic characteristics of the 327 patients were as follows: mean age 46.82±13.30 years ranging from 15 to 85; mean duration of illness 18.87±11.56; and mean GAF score of 41.82± 24.35. Most of the patients were unemployed (87%), with medical insurance (91%), and without family history of mental illness (81%); about half were male (49%), married (52%), and had an education level of primary school or below (47%).

Demographic characteristics of the 327 caregivers were as follows: mean age 57.68±12.54 years ranging from 17 to 81, with a 17-year duration of providing care, mean monthly household income was 1707 RMB/month, with a mean APGAR score of 6.29±3.25. Parents (46%) and spouse (35%) were the major type of kinship, accounting for 81% of the total caregivers. Most of the caregivers were married (84%), non-religious (82%) and with physical illness (67%). Over half were female (54%), employed (53%), with other dependents (55%) and co-caregivers (51%).

### Procedures

Ethics approval was granted by the Ethics Review Committee of the Xiangya School of Public Health of Central South University. The survey was conducted from November 2015 to January 2016. The interviewers were postgraduate and doctorate students in the social medicine department of the School of Public Health. All interviewers have received basic training in psychiatry during their first year study in the department and a one-week extensive training by two psychiatrists before the survey. All interviewers have participated in a one-month pilot study prior to the formal survey. During the survey, a regular meeting was hold every night to discuss cases and questionnairs to ensure consistency. In the first step, interviewers contacted the health center of each township, which provided us with one mental health specialist in charge of all registered patients in 686 Program of that township. In the second step, the township specialist introduced interviewers to all community/village doctors within the township. In the third step, each community/village doctor leads the interviewers to visit each household of all registered patients and explained the purpose and process of the study to the primary family caregivers. After providing written informed consent, each eligible respondent was invited to complete a series of questionnaires (see measures below) by face-to-face interviews. The answers were checked by a quality control person to ensure integrity, accuracy and consistency. All participants were reimbursed with some small gifts equivalent to RMB ¥ 10 (equal to USD$2) in return for their participation.

### Measures

#### Family burden

Family burden was assessed using the Family Burden Interview Schedule (FBIS). It is a 24-item survey classified into six categories viz., financial burden, disruption of routine family activities, family leisure, family interactions, and effect on physical and mental health of others [45]. Items are rated on a 3-point Likert scale from 0-“no burden,” 1- “moderate burden,” to 2-“serious burden”. The total score of FBIS ranges from 0 to 48 with higher scores indicating higher burden. A mean score of FBIS is obtained by dividing the total score of FBIS by the number of items, with a positive result defined as a mean score of ≥1, indicating moderate and severe burden [46]. Ever since its development in 1981, the instrument has been widely used and validated in numerous studies and in different countries including China [47–50]. In the present study, the Chinese version of FBIS showed acceptable internal consistency with a Cronbach’s α of 0.86.

#### Patient factor

Patient function was assessed using the Global Assessment of Functioning (GAF). It is one of the axes of the DSM-IV, with a 100‑point single‑item rating scale to assess patient’s function in three major domains during a particular time: Social functioning, occupational functioning, and psychological functioning [51]. The score of GAF ranges from 1–100, with higher score indicating higher function. Examples are given for each ten-level interval. GAF has proved to have satisfactory psychometric properties [51, 52]. The functional level of the patient over the past 1 month was assessed in this study with GAF.

Other patient factors selected in the present study included family history of mental illness, number of hospitalization, whether having medical insurance, illness duration, and medication adherence, which were all measured with one simple question in the self-designed information sheet.

#### Demographic factors

Demographic information included: 1) patient’s gender, age, ethnicity, education, occupation and 2) primary family caregiver’s gender, age, ethnicity, education, occupation, monthly household income, kinship with the patient, years of caring, whether having co-caregivers or other dependents, and whether having any physical illness.

#### Social psychology

Family function was assessed using the Family Adaptation, Partnership, Growth, Affection and Resolve Index scale (APGAR). It is a 5-item measure developed by Smilkstein [53] to assess caregiver’s satisfaction regarding their family functionality. Items are rated on a 3-point Likert scale from 0-‘‘hardly ever,” 1-‘‘sometimes,” to 2-‘‘almost always”. A total score of APGAR is obtained by adding up the scores of all 5 items and ranges from 0 to 10 with higher scores indicating a greater degree of satisfaction with family function. The APGAR has been widely used in various studies with satisfactory psychometric properties reported [54, 55]. Acceptable reliability was reached in the present study with a Cronbach’s alpha of 0.91.

Social network and support was measured using the self-designed Care Network Scale (CNS). It was originally developed based on the Norbeck Social Support Questionnaire (NSSQ) [56], and revised according to a pilot qualitative study on caregiving experience of family members of schizophrenia patients to make it more targeted to the present study population. The CNS consists of four questions covering four aspects of caregiving including daily care, medicine management, medical care and financial care. To complete the CNS, participants firstly listed the names of people who provided the patient with care. They were then asked to rate the frequency of each of the four kinds of care on a 5-point Likert-type scale ranging from 0 (never) to 4 (always) that is available from each of the names on their list. A total care score was generated by summing the scores of all caregivers on the list, with higher score indicating more care and support. Detailed information about the questions and optional answers can be seen in the S1 File.

### Statistical analysis

Data were analyzed using STATA software version 12.0. Data were examined for the presence of missing values, influential values and outliers, skew, and kurtosis. Scales and indices were tested for reliability. Exploratory and summary statistics were obtained for all variables within the dataset. Both univariate and multivariate logistic regression analyses were conducted to examine predictors of family burden.

## Results

### Level and contents of family burden

Table 1 presents the burden level and the contents of the burden of the family. The mean score of FBIS was 23.62±9.76 (range, 0–48), with positive rate of 52%, indicating over half caregivers reported their family burden being moderate and severe. Among the six dimensions of family burden, financial burden was the most frequently reported one with a positive rate of 76%, followed by effect on mental health of others (70%), disruption of routine family activities (58%), disruption of family leisure (50%), effect on physical health of others (48%). Disruption of family interactions (37%) was the least mentioned type of burden. A further paired t-test was used to compare the mean scores of six subscales of family burdens, which also found that the mean score of financial burden was higher than that of the other five types of burden (results not shown here). Thus, financial burden was the most significant burden in both mean score and positive rates among the six subscales of family burden.

**Table 1:** Level and contents of family burden (n = 303).

Questions	Mean score M(SD)	Positive rate N (%)	Rank
**Financial burden**	**1.24(0.50)**	**229(75.58)**	**1**
1. Loss of patient’s income	1.30(0.90)	214(70.63)	8
2. Loss of income of other family members	1.44(0.76)	253(83.50)	2
3. Expenses of patient’s illness	1.49(0.71)	264(87.13)	1
4. Expenses due to other necessary changes in arrangements	0.71(0.86)	134(44.22)	22
5. Loans taken	1.19(0.86)	216(71.29)	7
6. Any other planned activity needing finance, postponed	1.34(0.81)	238(78.55)	4
**Disruption of routine family activities**	**1.03(0.54)**	**176 (58.09)**	**3**
7. Patient not attending work, school, etc.	1.40(0.83)	235(77.56)	5
8. Patient unable to help in household duties	1.19(0.87)	213(70.30)	9
9. Disruption of activities due to patient’s illness and care	1.01(0.86)	194(64.03)	11
10. Disruption of activities due to patient’s irrational demands	0.82(0.86)	159(52.48)	15
11. Other family members missing school, meals, etc.	0.71(0.80)	148(48.84)	17
**Disruption of family leisure**	**0.88(0.62)**	**152 (50.17)**	**4**
12. Stopping of normal recreational activities	0.78(0.84)	155(51.16)	16
13. Absorption of another member’s holiday and leisure time	1.09(0.83)	211(69.64)	10
14. Lack of participation by patient in leisure activity	0.91(0.88)	173(57.10)	14
15. Planned leisure activity abandoned	0.73(0.83)	145(47.85)	18
**Disruption of family interactions**	**0.75(0.57)**	**112(36.96)**	**6**
16. Effect on general family atmosphere	1.23(0.82)	229(75.58)	6
17. Other members arguing over the patient	0.68(0.83)	135(44.55)	20
18. Reduction or cessation of interaction with friends and neighbors	0.72(0.84)	143(47.19)	19
19. Family becoming secluded or withdrawn	0.50(0.77)	102(33.66)	24
20. Any other effect on family or neighborhood relationships	0.61(0.81)	122(40.26)	23
**Effect on physical health of others**	**0.76(0.70)**	**144(47.52)**	**5**
21. Physical illness in any family member	0.67(0.82)	135(44.55)	21
22. Any other adverse effect on others	0.85(0.81)	179(59.08)	13
**Effect on mental health of others**	**1.13(0.74)**	**212(69.97)**	**2**
23. Any member seeking professional help for psychological illness	0.95(0.87)	180(59.41)	12
24. Any member becoming depressed, weepy, irritable	1.31(0.79)	238(79.54)	3
Total	0.98(0.41)	158(52.15)	

The first three rankings of burden were ‘Expenses of patient’s illness’ (87%), ‘Loss of income of other family members’ (84%), and ‘Any member becoming depressed, weepy, irritable’ (80%); while the last three rankings of burden were ‘Expenses due to other necessary changes in arrangements’(44%), ‘Any other effect on family or neighborhood relationships’(40%), and ‘Family becoming secluded or withdrawn’(34%).

### Predictors of family burden

The results of a univariate logistic regression of the effect of disease-related factors, demographics, and social psychological factors on family burden are presented in Table 2. Factors that were significantly associated with family burden in univariate logistic regression were: number of admissions, patient function, patient age, caregiver gender, patient marriage, patient education, caregiver education, caregiver’s additional dependents, caregiver’s physical illness, caregiver’s kinship with the patient, family function and care network function. Patient having more admissions, caregiver being female, patient being unmarried, patient having middle school education, caregiver having additional dependents, caregiver being parents, and a higher care network function were associated with increased family burden; while higher patient function, increasing patient age, caregiver having high school education, and higher family function were associated with a decreased family burden.

**Table 2:** Univariate logistic analyses of disease-related factors, demographics, social psychology, and family burden (n = 303).

Variable	Family burden		OR(95% CI)	p-value
	Negative (n = 145)^a^	Positive (n = 158)^a^		
Patient-related factors				
Family history				
No	121(83.45)	123(77.85)	ref	
Yes	24(16.55)	35(22.15)	1.43(0.81, 2.55)	0.219
Number of admissions				
<3	82(58.16)	62(39.74)	ref	
≧3	59(41.84)	94(60.26)	2.11(1.33, 3.35)	0.002
Insurance				
No	130(89.66)	145(91.77)	ref	
Yes	15(10.34)	13(8.23)	0.78(0.36, 1.69)	0.525
Illness duration	19.49±0.95	18.23±0.95	0.99(0.91, 1.01)	0.3527
Medication adherence ^b^	24.62±0.90	24.98±0.86	1.00(0.98, 1.02)	0.7714
GAF score	49.55±1.97	34.41±1.81	0.97(0.96, 0.98)	0.0000
(patient function)				
Demographic factors				
Location				
Yutan	24(16.55)	30(18.99)	ref	
Chengjiao	23(15.86)	29(18.35)	1.01(0.47, 2.17)	0.982
Liushahe	75(51.72)	75(47.47)	0.80(0.43, 1.49)	0.484
Shuangfupu	23(15.86)	24(15.19)	0.83(0.38, 1.83)	0.652
Patient age	50.34±1.06	43.5±1.06	0.96(0.94, 0.98)	0.0000
Caregiver age	57.63±1.15	57.66±0.89	1.00(0.98, 1.02)	0.984
Patient gender				
Male	68(46.90)	84(53.16)	ref	
Female	77(53.10)	74(46.84)	0.78(0.50, 1.22)	0.276
Caregiver gender				
Male	74(51.03)	62(39.24)	ref	
Female	71(48.97)	96(60.76)	1.61(1.02, 2.55)	0.039
Patient marriage				
Married	83(57.24)	71(44.94)	ref	
Unmarried ^c^	62(42.76)	87(55.06)	1.64(1.04, 2.58)	0.032
Caregiver marriage				
Married	121(83.45)	132(83.54)	ref	
Unmarried ^c^	24(16.55)	26(16.46)	0.99(0.54, 1.82)	0.982
Patient occupation				
Employed	23(15.86)	18(11.39)	ref	
Unemployed	122(84.14)	140(88.61)	1.47(0.76, 2.85)	0.256
Caregiver occupation				
Employed	78(53.79)	81(51.27)	ref	
Unemployed	67(46.21)	77(48.73)	1.11(0.70, 1.74)	0.660
Patient education				
Primary	76(52.41)	64(40.51)	ref	
Middle	45(31.03)	65(41.14)	1.72(1.04, 2.84)	0.036
High	24(16.55)	29(18.35)	1.43(0.76, 2.71)	0.265
Caregiver education				
Primary	84(57.93)	96(60.76)		
Middle	34(23.45)	47(29.75)	1.21(0.71, 2.05)	0.481
High	27(18.62)	15(9.49)	0.49(0.24, 0.97)	0.042
Monthly household income (RMB/month)				
<1,000	57(39.58)	77(49.68)	ref	
≧1,000	87(60.42)	78(50.32)	0.66(0.42, 1.05)	0.079
Co-caregivers ^d^				
No	74(51.03)	71(44.94)	ref	
Yes	71(48.97)	87(55.06)	1.28(0.81, 2.01)	0.288
Additional dependents				
No	77(53.10)	56(35.44)		
Yes	68(46.90)	102(64.56)	2.06(1.30, 3.27)	0.002
Physical illness				
No	56(38.62)	40(25.32)	ref	
Yes	89(61.38)	118(74.68)	1.86(1.14, 3.03)	0.013
Kinship with the patient				
Spouse	55(39.01)	46(29.49)	ref	
Parents	54(38.30)	87(55.77)	1.93(1.15, 3.23)	0.013
Siblings	13(9.22)	12(7.69)	1.10(0.46, 2.65)	0.825
Children	19(13.48)	11(7.05)	0.69(0.30, 1.60)	0.390
Length of caring	17.54±0.98	16.62±0.79	0.99(0.97, 1.01)	0.459
Social psychology				
Family function	6.97±0.24	5.66±0.28	0.88(0.82, 0.95)	0.001
(APGAR score)				
Care network size	3.64±0.13	3.79±0.11	1.07(0.92, 1.25)	0.3847
Care network function (CNS score)	15.51±0.70	19.41±0.71	1.05(1.02, 1.08)	0.0001

^a^ means n (%) if not otherwise indicated.

^b^ Medication adherence was assessed by one simple question of “during the past one month, how many days have the patient taken medicines as suggested?”

^c^ Unmarried includes single, divorced or widowed

^d^ Co-caregiver refers to a person who was also involved in the caregiving activities besides the primary family caregivers.

A further multivariate logistic regression was conducted to determine the predictors of family burden. Among the 26 factors that were included in the model, eight factors remained significant after controlling for all the other factors, with four categorical variables(that is, number of admissions, caregiver’s gender, education, and additional dependents) and four continuous variables(that is, patient’s function and age, family function, and care network function) (Table 3). Among the 4 categorical predictors of family burden, patient being admitted for over 3 times, caregiver being female, having a middle school education, and with additional dependents were over twice as likely to have a moderate and above family burden as their counterparts, with an odds ratio ranging from 2.38 to 2.83. Among the 4 continuous predictors of family burden, every one point increase in patient function and family function, or one year increase in patient age will decrease the positive rate in family burden by 3%, 14% and 6%, respectively; while every one point increase in the care network function score will increase the positive rate in family burden by 5%. Among all predicting factors, the number of admissions was the strongest categorical predictor of family burden, while family function was the strongest continuous predictor.

**Table 3:** Multivariate logistic regression of disease-related factors, demographics, social psychology, and family burden (n = 303)*.

Variable	Family burden	
	adjusted *OR* (95% CI)	*P* value
**Number of admissions**		
<3	ref	
≧3	**2.83(1.44, 5.56)**	0.003
**Caregiver gender**		
Male	ref	
Female	**2.38(1.10, 5.12)**	0.027
**Caregiver education**		
Primary	ref	
Middle	**2.40(1.07, 5.40)**	0.035
High	0.55(0.19, 1.57)	0.261
**Additional dependents**		
No	ref	
Yes	**2.40(1.18, 4.90)**	0.016
**Patient function(GAF)**	**0.97(0.96, 0.99)**	0.000
**Patient age**	**0.94(0.89, 0.99)**	0.036
**Family function**	**0.86(0.77, 0.96)**	0.008
**Care network function**	**1.05(1.01, 1.09)**	0.027

*Family history of mental illness, whether having insurance, illness duration, medication adherence, location, caregiver age, patient gender, patient marriage, caregiver marriage, patient occupation, caregiver occupation, patient education, caregiver household income, whether having co-caregivers, caregiver having physical illness, kinship with the patient, length of care, caregiving network size were not significantly associated with family burden and thus are not listed in this table

## Discussion

### Summary of the findings

China has experienced tremendous social changes in the past three decades, including demographic, socioeconomic, sociocultural and epidemiological changes [57]. All these changes have brought about the gradual collapse of the traditional model of caring for mental patients by family members. Fewer and fewer family members were willing to and capable of caring for their mentally sick relatives. There is a high possibility that a growing number of mental patients will be left unattended in the future. It is thus crucial and urgent to understand the family burden these caregivers face and its associated factors.

The main finding of the study are that the mean score of FBIS was 23.62±9.76, with over half (52%) caregivers reported their family burden being moderate and severe. Among the six domains of family burden, financial burden (76%) was the most common burden, followed by effect on mental health of others, disruption of routine family activities, disruption of family leisure, effect on physical health of others, while disruption of family interactions (37%) was the least mentioned. Patient being admitted for over 3 times, caregiver being female, having a middle school education, and with additional dependents, as well as higher care network function were positive predictors of family burden, while higher patient function and family function, and increasing patient age were negative predictors of family burden. The number of admissions was the strongest categorical predictor of family burden, with an OR of 2.83(95% CI: 1.44–5.56); while family function was the strongest continuous predictor with an OR of 0.86(95% CI: 0.77–0.96).

### Level and contents of family burden

Over half (52%) caregivers reported their family burden being moderate and above, with a total mean score of 23.62 ± 9.76 in the total study population. The score in the present study was lower than that reported in Fallahi’s study of an Iranian sample (40.64 ± 2.88/40.51 ± 3.17) [58] and Koujalgi’s study of a India sample(29.96 ±7.00) [59]; similar to that reported in Lasebikan’s study of a Nigeria sample (22.69 ±6.21) [60] and Thomas’s study of another India sample (21.74 ± 7.50) [61], but much higher than that reported in Chan’s study in a Hongkong sample (15.75±9.27/ 12.54 ±8.43) [25]. When compared within mainland China, the family burden score in the rural Ningxiang County of Hunan province was comparable to most areas in China including Changsha city of Hunan province [62, 63], Shandong province [63, 64], Shanghai [65, 66], and Beijing [67], indicating that our results are similar to the average national level of family burden for schizophrenia patients.

However, the ranking of the six burden domains in the present study shows variance with other studies in China. In the present study, financial burden was ranked as the highest and family interaction was rated as the lowest, which is different with most other studies ranking routine activities disruption and family interaction as the highest [46, 67, 68] and physical health and mental health as the lowest[46, 67–69]. The divergence may be explained by the specific economic and cultural characteristics of our study site. The rural areas of Ningxiang County were mainly agricultural regions, where most families depend on farming for life and can only be self-sustained, having a patient with schizophrenia means significantly increased direct and indirect medical costs, as well as markedly decreased labor productivity from both the patient and the caregivers [4], which will result in tremendous financial burden to the family, as exemplified by the low family income and high medical expenses in the present study(results not shown). On the other hand, the rural areas are deeply rooted in the traditional confusion culture that attach great importance to family harmony and cohesion [9, 11]. When confronted with disease in one family member, it is incumbent on the whole family to hold together to take good care of the sick one and to keep intimate kinship ties as well as warm family atmosphere. As a result, family interaction would be least affected by a schizophrenia patient in the present study.

### Predictors of family burden

#### Patient factor

Higher patient function negatively predicted family burden, which was consistent with existing literature [10, 15, 19]. The result is self-explanatory considering that managing the ill behaviors and keeping the normal function of patients have always been the main focus and major needs of family caregivers [19]. The result that family with a patient being admitted for over 3 times was a strongest predicting factor of family burden was a novel finding that has scantly been reported in other studies. The most likely reason may be that more admissions always reflect worse patient function, which will directly lead to increased family burden. Economy may be an additional explanation, especially in this rural population with low socio-economic status. More patient admissions are often followed by huge expenses on treatment and caring, which was further proved by the fact that financial burden was the most frequently mentioned burden in this study. Another possible reason may be that schizophrenia patients in hospitalization were often worse treated and less adequately cared for than patients at home, as reported by both patients and primary family caregivers during our qualitative interviews, this will bring about more concern and anxiety among caregivers, which will in turn produce higher family burden. One implication of this finding was to explore an alternative care model that is more cost-effective and suitable to both patients and family caregivers than the current hospitalization care. Various successful community programs targeted at alleviating family burden have already been reported in other parts of the world [70–73], with plenty of good experiences for China to learn from.

#### Demographic factors

The finding that patient age, caregiver gender, caregiver education and additional dependents were predictors of family burden also finds its support in various studies, indicating the need of full assessment of family’s socio-demographic characteristics in planning targeted family intervention to alleviate family burden.

The study shows that families with younger patients and additional dependents experienced higher burden than their counterparts, which is consistent with most findings [29, 74, 75]. Younger patient means more demands for attention and care, as well as more life-lost productivity to the family, while additional dependents requires more investment of time and energy from caregivers on caring for multiple dependents, both will result in higher family burden.

Female caregivers were more likely to have family burden than males, which is also in accordance with the literature [74, 76, 77]. According to the World Federation of Mental Health [78], the majority of caregiving task are shouldered by women, with an estimated proportion up to 80%. Those women are usually in a disadvantaged socio-economic position with less education and lower income, and have less resource to deal with the challenges of intensive caregiving. As a result, the perceived burden can be tremendous, as exemplified by some studies showing a 5-time fold increase in the risk of having depression and anxiety among female caregivers [78]. This suggests that more support should be provided to female caregivers in understanding their concerns and meeting their needs. Another implication of this finding is the potential to involving more males in caregiving tasks, which means a reversal of traditional gender roles within family and warrants additional research in the future [9].

In contrast to most studies indicating a negative predicting effect of higher education in caregivers on family burden [29, 76], the association between caregiver education and family burden was not presented as a straight line, but as a reversed “U-shape”, that is, family burden increase with middle school education, but then decrease with high school education and above. One possible reason may be that, as compared to the caregivers with primary school education and below who have less knowledge and thus lower level of fear, worry and shame related to schizophrenia, those with middle school education have known more about schizophrenia, which may bring about more horror and stigma on the family, and thus lead to more burden [79]. However, when the caregivers’ education levels increase to high school and above, the stigma is mitigated by socio-economic advantages and better resources of managing the disease brought about by much higher education, thus leading to a decreased family burden. Further investigation of caregiver’s education on family burden is warranted.

#### Social psychological factors

In terms of social psychological factors, higher family function was the strongest continuous negative predictor for family burden, which is supported by abundant studies [38, 39, 80]. A well-functional family often means more strength and resources within family, making it less vulnerable and more resistant to the excessive stress and strains confronted by the family [74]. This result suggests that future research and programs aimed at alleviating family burden may benefit most from targeting at improving family function.

Contrary to most studies showing a positive association between social network, social support and family burden [4, 33–35], the present study finds no significant association between family burden and care network size, and presents negative association between family burden and care network function. One possible reason of the unexpected negative association may be a focus on measuring objective social network function rather than the subjective social support of the CNS scale. As shown in the CNS, all items asked about the actual involvement of specific daily caregiving tasks of family members instead of their perceived support. It is likely that higher CNS scale implies more involvement of caregiving tasks, which may lead to higher family burden due to the burning out of family members in caring for the patient. On the other hand, more involvement of caregiving activities within family may indicate lower social support from outside the family, which may also lead to increased family burden. More research is needed to compare the differences of the CNS scale and standard social support scale in measuring various dimensions of social support, as well as to explore the relationship between CNS and family burden.

One major limitation of the present study is the adaptability and applicability of the FBIS in the present study to measure family burden. As a complex and multidimensional construct that defies a uniform and standard definition, family burden covers a broad range of scope involving emotional, social, financial or physical investment and psychological experiences in reaction to caregiving process that goes beyond a simple operationalization and measurement [4]. Although the development of FBIS is intended to measure the various dimensions of family burden, it is still relatively inadequate with regards to touching on every specific aspect related to family burden. The FBIS was originally developed by Pai and Kapur’s in an India population through free unstructured interviews[45]. It is likely that some important information on family burden may be missing during the interview, information extraction and item simplification process. For instance, we are not able to measure the concrete financial cost including both direct and indirect cost of the treatment and care of schizophrenia patients from the FBIS, thus we cannot make an objective assessment of the economic burden of the disease on the family; additionally, some other potential costs are not measured from the FBIS, among which the most significant one is the opportunity loss of caregivers initiated by caring for the schizophrenia patients. With all this potential missing information, family burden based on FBIS may be underestimated. A comprehensive assessment of family burden by combining the FBIS with other scales covering information on other costs, or by adding an in-depth qualitative interview to the FBIS may be warranted in the future research. Another limitation is the cross-sectional design of the study making it impossible to make an accurate assessment of the family burden during the course of caregiving, which is an evolving and dynamic process. Future research may benefit from adding a qualitative study and developing new measurement tools and methods to measure family burden.

## Conclusion

In conclusion, this study represents an attempt to better understand the family burden in rural Chinese communities. Family burden of schizophrenia patients in the present study was comparable to other parts of China. Financial burden is the most significant burden mentioned by the families, confirming the importance and need to increase and strengthen economic support for families inflicted by schizophrenia. Furthermore, the positive relationship between patient’s admissions, caregiver’s gender, education, additional dependents and family burden; as well as the negative relationship between patient function, family function and family burden was informative. Future intervention toward alleviating family burden may be best served by improving family function and exploring alternative care model instead of hospitalization. Special attention should also be paid to family’s socio-demographic characteristics in planning targeted intervention to alleviate family burden

## Supporting information

S1 File Appendix caregiving experience. (DOCX)
