# Supplementary material for: Reported family burden of schizophrenia patients in rural China
Source: PLoS One. 2017 Jun 19;12(6):e0179425. doi: 10.1371/journal.pone.0179425 (PMC5476254; doi:10.1371/journal.pone.0179425)
Supplement: S1 File — (DOCX) [file pone.0179425.s001.docx]

**Appendix 1 Caregiving experience**

- 1. Are you involved in taking care of the patient’s daily activities, such as eating, drinking, getting dressed, getting a shower, going to the toilet, getting outside?

① Yes ② No

- 1. If yes, how often are you involved in taking care of the patient’s daily activities?

① Occasionally ② Sometimes ③ Often ④ always

- 1. Are you involved in managing the patient’s medicine, such as monitoring medication, helping with buying and getting the medicine, etc.?

① Yes ② No

2.1 If yes, how often are you involved in managing the patient’s medicine?

① Occasionally ② Sometimes ③ Often ④ always

- 1. Are you involved in helping with the patient’s hospital visit, such as taking the patient to the doctor, helping with register, hospitalization, etc.?

① Yes ② No

3.1 If yes, how often are you involved in helping with the patient’s hospital visit?

① Occasionally ② Sometimes ③ Often ④ always

- 1. Are you involved in providing financial support to the patient’s, such as spending money on the patient, giving money to the patient, etc.?

① Yes ② No

4.1 If yes, how often are you involved in providing financial support to the patient?

① Occasionally ② Sometimes ③ Often ④ always
